# Cyanide poisoning in inhalation injuries

**DOI:** 10.1002/ccr3.3099

**Published:** 2020-08-07

**Authors:** Sharon Kennedy, Kevin C. Cahill

**Affiliations:** ^1^ National Burns Unit Department of Plastic and Reconstructive Surgery St. James’s Hospital Dublin Ireland

**Keywords:** critical care medicine, emergency medicine, endocrinology and metabolic disorders, general surgery

## Abstract

Cyanide gas forms during the combustion of synthetic polymers and should be considered in patients presenting with inhalation injuries. A persistently high lactate following adequate resuscitation may be an indicator of cyanide exposure. As cyanide poisoning can be rapidly fatal, prompt recognition and treatment of this condition is vital.

## Graphical abstract

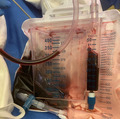

A 78‐year‐old man was admitted to a National Burns Unit following a 22% total body surface area flame burn and inhalation injury. This occurred following an explosion while lighting a gas fire in his outhouse. Despite adequate fluid resuscitation and good baseline renal function, a severe increased anion gap metabolic acidosis, with an associated elevated lactate (2.26 mmol/L), persisted. Cyanide poisoning was suspected, and hydroxocobalamin was administered. Following administration, his urine rapidly turned a characteristic red‐wine color (Figure 1). Cyanide is a mitochondrial toxin which preferentially binds ferric ions in cytochrome oxidase a_3_—inhibiting this final enzyme in the mitochondrial cytochrome complex. This causes oxidative phosphorylation to cease. Cells switch to anaerobic metabolism leading to the formation of lactic acid and a metabolic acidosis.^1^ Hydroxocobalamin is a synthetic form of vitamin B12 which binds cyanide and forms the nontoxic cyanocobalamin. This is renally cleared, giving the urine a dark red color. Onset of chromaturia typically occurs within the first 2 hours following administration and can persist for up to 35 days.^2^

**Figure 1:**
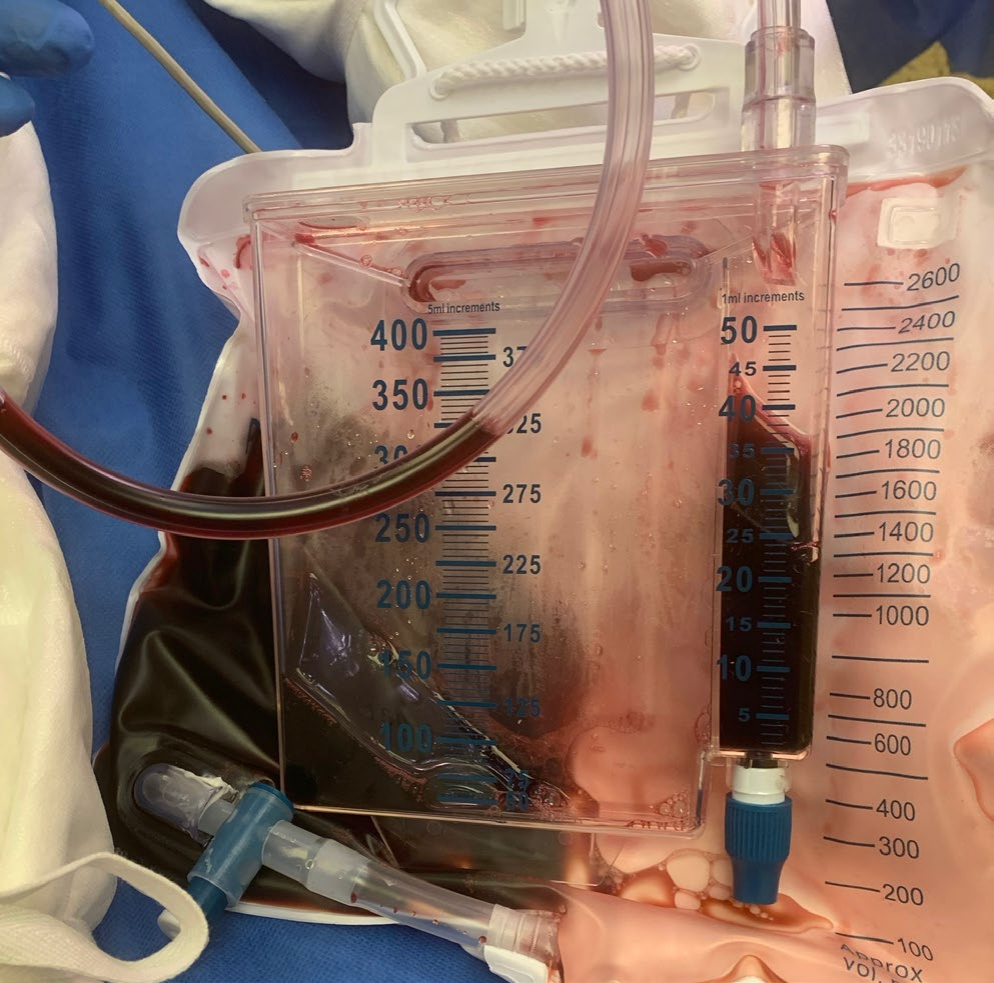
Red‐wine colored urine as a result of hydroxocobalamin administration

Cyanide gas forms during the combustion of synthetic polymers often found in building materials and furnishings. As cyanide gas can be rapidly fatal, a low threshold for treatment should exist in those suspected of having inhalation injuries.

Within 7 hours of administration of hydroxocobalamin, the patient's acidosis had resolved and his lactate had significantly improved (1.49 mmol/L). As expected, his urine remained discolored for approximately three weeks. After a protracted hospital stay, the patient was discharged home well and has since returned to work in his family business.

## CONFLICT OF INTEREST

None declared.

## AUTHOR CONTRIBUTIONS

SK: drafted and reviewed the article. KC: reviewed the article.

## ETHICAL APPROVAL

The regional Research Ethics Committee judged that this work was exempt from ethical review.
